# Clinical outcomes of frozen autograft reconstruction for the treatment of primary bone sarcoma in adolescents and young adults

**DOI:** 10.1038/s41598-021-96162-5

**Published:** 2021-08-27

**Authors:** Yoshihiro Araki, Norio Yamamoto, Katsuhiro Hayashi, Akihiko Takeuchi, Shinji Miwa, Kentaro Igarashi, Takashi Higuchi, Kensaku Abe, Yuta Taniguchi, Hirotaka Yonezawa, Sei Morinaga, Yohei Asano, Hiroyuki Tsuchiya

**Affiliations:** Department of Orthopaedic Surgery, Graduate School of Medical Sciences, Kanazawa University, 13-1, Takaramachi, Kanazawa, Ishikawa 920-8641 Japan

**Keywords:** Oncology, Cancer, Surgical oncology, Bone cancer, Sarcoma

## Abstract

Age affects the clinical outcomes of cancer treatment, including those for bone sarcoma. Successful reconstruction using frozen autograft after excision of bone sarcoma has been reported; however, little is known about the clinical outcomes of frozen autograft reconstruction according to age. The purpose was to evaluate the clinical outcomes of the frozen autograft reconstruction focusing on skeletally mature adolescents and young adults (AYAs) that was 15 to 39 years of age. A total of 37 AYA patients with primary bone sarcoma on the appendicular skeleton were enrolled in this study. The mean follow-up period was 89 months. The graft survival (GS), overall survival (OS), recurrence-free survival (RFS), complications and the function were retrospectively evaluated using medical records. The 10-year GS, OS, and RFS rates were 76%, 84%, and 79%, respectively. Bone union was achieved with a rate of 94% within 1 year after surgery, and nonunion (n = 1) and fracture (n = 2) were infrequently observed. Graft removal was performed in 7 cases, and the most common reason for the removal was infection (n = 5). The Musculoskeletal Tumor Society score was excellent in 23 cases of the available 29 cases. Frozen autograft reconstruction for AYAs showed excellent clinical outcomes, although the long-term follow-up is required.

Bone sarcoma often occurs in children, adolescents and young adults (AYAs)^1–3^. In contrast to pediatric patients, the bone in AYA patients is skeletally mature because the physis has disappeared due to the completion of the growth. After growing up and having completed their psychosocial development, AYAs play outside less frequently than children, and they often do sports activities, drive, work, and raise a family, which are natural activities in daily living^2^.

The mainstay of the treatment for bone sarcoma is surgery with chemotherapy^3,4^. Amputation is a radical treatment; however, severe impairment of daily living remains, and limb salvage surgery is generally performed for bone sarcoma in order to allow the patient to continue a normal life^5–8^. Limb salvage surgery includes tumor excision and reconstruction with bone graft, synthetics, or an implant, and there are several reconstruction techniques. Reconstruction using a tumor prosthesis is commonly performed after excision of bone tumors in all generations^9,10^. However, this type of reconstruction has a limited durability and several reoperations will be necessary, especially for young patients. The reasons for revision surgery include mechanical failure, loosening, chronic infection, and other conditions^9–12^.

Reconstruction using an allograft or autograft is a biological procedure after bone tumor excision. However, the use of allografts is not widespread in Japan due to either ethical reasons or a lack of easy availability^13–16^. On the other hand, devitalized autograft reconstruction received coverage by Japan’s social insurance system in April 2020, and these procedures can be performed at any hospital in Japan. Different types of devitalized autograft are used for reconstruction, including irradiated bone^17–20^, pasteurized bone^21–23^, and frozen bone^24–32^. Autograft reconstruction has been reported to be associated with numerous advantages: the autograft can fit perfectly with the original site, and the retracted tendon or muscles can be reattached, even in cases where autograft-prosthesis composite fixation is performed^29,30^.

When managing patients with bone tumors, consideration of the impact on the bone properties is essential for obtaining good clinical outcomes and reducing the rates of complications associated with bone properties. However, little is still known about the bone properties of devitalized autograft according to age, and clinical outcomes and complications of autograft reconstruction according to age were not sufficiently investigated.

Several complications are inevitable as follows: non-union, infection, fracture, and implant failure related to the surgical treatment of bone tumors^17–32^, however, surgeons should fully understand both the advantages and disadvantages of such surgical procedures according to the patient’s age. For AYA patients who participate in social activities, the longevity of normal limb function and achieving a successful cure without debilitating problems due to late toxicities assoicated with surgery should be considered as the main treatment goal^2–4^. We therefore investigated the clinical outcomes and complications of frozen autograft reconstruction in AYAs, and compared with those in children and older adult patients.

## Results

### Patient characteristics

The study population in the AYA groups included 20 male patients and 17 female patients (mean age, 23 years; range, 15–39 years). The mean follow-up period was 89 months (range, 5–229 months). The tumor locations included the upper extremities (proximal humerus; n = 5) and lower extremities (femur [proximal; n = 3, diaphysis; n = 1, distal; n = 16], tibia [proximal; n = 7, diaphysis; n = 2, distal; n = 2], and calcaneus [n = 1]). Involvement of the physis was observed in 8 cases. The histological types according to the 2013 WHO classification^1^ included osteosarcoma (n = 29), Ewing sarcoma (n = 3), undifferentiated pleomorphic sarcoma of bone (n = 3), chondrosarcoma (n = 1), malignant hemangiopericytoma (n = 1). Staging according to the AJCC 8th classification^33^ were stage IIA (n = 12), stage IIB (n = 14), stage III (n = 2), stage IVA (n = 2), and stage IVB (n = 7). Pedicle freezing and free freezing procedures were applied for 16, and 21 cases, respectively. Intercalcary fixation, osteoarticular fixation, and prosthesis-composite fixation after freezing procedures were performed as reconstruction methods in 11, 10, and 16 cases, respectively. The patient charatcteristics were investigated separately and compared in the three different age groups, and there were no significant differences in patients characteristics regarding sex, follow-up period, tumor location, staging, freezing procedure, or reconstruction method in the three age groups (Table 1). Regarding histological subtype, osteosarcoma showed a lower rate in older adults than in children and AYA patients, but, the histology in all cases in the present study was primary bone sarcoma.

**Table 1:** The demographic data of the patients who underwent frozen autograft reconstruction on the appendicular skeleton in the three age groups.

Generation	Children (≤ 14 years old)	AYAs (15 to 39 years old)	Older adults (≥ 40 years old)	*P*-value
Cases (n)	27	37	16	–
Median age (years old) (range)	11 (6–14)	23 (15–39)	59 (41–76)	< 0.01
Sex (Male: Female)	15:12	20:17	10:6	0.92
Median follow-up periods (mos.) (range)	87 (3–237)	89 (5–229)	86 (26–163)	0.98
Stage (AJCC8th) (n) (%)	IIA 2 (7%) IIB 18 (67%) III 2 (7%) IVB 5 (19%)	IIA 12 (32%) IIB 14 (38%) III 2 (5%) IVA 2 (5%) IVB 7 (19%)	IIA 6 (38%) IIB 8 (50%) III 1 (6%) IVB 1 (6%)	0.08
Tumor location (n) (%)	Upper extremities 2 (7%) Lower extremities 25 (93%)	Upper extremities 5 (14%) Lower extremities 32 (86%)	Upper extremities 4 (25%) Lower extremities 12 (75%)	0.30
Histological types (n) (%)	Osteosarcoma 25 (93%) Ewing sarcoma 2 (7%)	Osteosarcoma 29 (78%) Ewing sarcoma 3 (8%) UPS 3 (8%) Chondrosarcoma 1 (3%) Malignant hemangiopericytoma 1 (3%)	Osteosarcoma 8 (50%) Chondrosarcoma 4 (25%) Fibrosarcoma 2 (13%) Adamantinoma 1 (6%) Malignant PMT 1 (6%)	< 0.01
Freezing procedures (n) (%)	Free freezing 11 (41%) Pedicle freezing 16 (59%)	Free freezing 21 (57%) Pedicle freezing 16 (43%)	Free freezing 5 (31%) Pedicle freezing 11 (69%)	0.21
**Reconstruction methods (n) (%)**				
Intercalary	16 (59%)	11 (30%)	7 (44%)	0.16
Osteoarticular	5 (19%)	10 (27%)	2 (13%)	
Composite	6 (22%)	16 (43%)	7 (44%)	

*AJCC* American Joint Committee on Cancer, *UPS* undifferentiated pleomorphic sarcoma, *PMT* phosphaturic mesenchymal tumor, *mos* months.

### The oncological outcomes

The chemotherapy was performed in 35 AYA patients (95%; 35/37), while 100% (27/27) in pediatric patients and 50% (8/16) in older adult patients underwent chemotherapy, respectively (*p* < 0.01). In some older adult patients, chemotherapy could not be performed due to a histology that was not chemosensitive, such as chondrosaroma and adamantinoma, or due to a high risk of adverse events in consideration of their advanced age. At the final follow-up examination, the oncological outcomes were clinically disease-free (n = 16), no evidence of diseases (n = 6), alive with disease (n = 5), and dead due to disease (n = 10), which revealed no significant differences in comparison to the other two age groups (Table 2).

**Table 2:** The clinical outcomes and complications in the patients who underwent frozen autograft reconstruction on the appendicular skeleton in the three age groups.

Generation	Children (≤ 14 years old)	AYAs (15 to 39 years old)	Older adults (≥ 40 years old)	*P*-value
Case (n)	27	37	16	–
Chemotherapy (n)	27	35	8	< 0.01
Oncological outcome (n) (%)	CDF 16 (59%)	CDF 16 (43%)	CDF 10 (63%)	0.58
	NED 5 (19%)	NED 6 (16%)	NED 1 (6%)	
	AWD 2 (7%)	AWD 5 (14%)	AWD 3 (19%)	
	DOD 4 (15%)	DOD 10 (27%)	DOD 1 (6%) DOOD 1 (6%)	
Median period of bone union (mos.) (range)	6 (3–15)	8 (3–21)	10 (4–24)	0.14
Bone union rate within 1 year	94%	94%	63%	0.06
Functional score (MSTS score) (n) (%)	Excellent 18 (86%) Good 2 (10%) Fair 1 (5%) Poor 0 (0%)	Excellent 23 (79%) Good 4 (14%) Fair 2 (7%) Poor 0 (0%)	Excellent 13 (87%) Good 1 (7%) Fair 1 (7%) Poor 0 (0%)	0.98
5-year graft survival (%)	80%	86%	79%	0.79
**Reasons for graft removal**				
Infection (n) (%)	1 (4%)	5 (14%)	3 (19%)	0.28
Fracture (n) (%)	1 (4%)	0 (0%)	0 (0%)	0.54
Non-union (n) (%)	0 (%)	0 (0%)	0 (0%)	1
Osteoarthritic change (n) (%)	2 (7%)	0 (0%)	0 (0%)	0.15
Recurrence (n) (%)	3 (11%)	2 (5%)	1 (6%)	0.86
**Complications**				
Infection (n) (%)	1 (4%)	7 (19%)	3 (19%)	0.17
Fracture (n) (%)	8 (30%)	2 (5%)	1 (6%)	0.03
Non-union (n) (%)	3 (11%)	1 (3%)	1 (6%)	0.35
Limb deformity (n) (%)	5 (19%)	1 (3%)	0 (0%)	0.05
Osteoarthritic change (n) (%)	4 (15%)	5 (14%)	0 (0%)	0.37
Bone absorption (n) (%)	1 (4%)	3 (8%)	2 (13%)	0.56
**Salvage amputation**				
Recurrence (n) (%) Infection (n) (%)	2 (7%) 1 (4%)	2 (5%) 3 (8%)	1 (6%) 0 (0%)	1 0.67

*CDF* clinically disease-free, *NED* no evidence of disease, *AWD* alive with disease, *DOD* dead of disease, *DOOD* dead of other diseases, *MSTS* Musculoskeletal Tumor Society.

### The grafat survival (GS) and oncological survival

**Figure 1:**
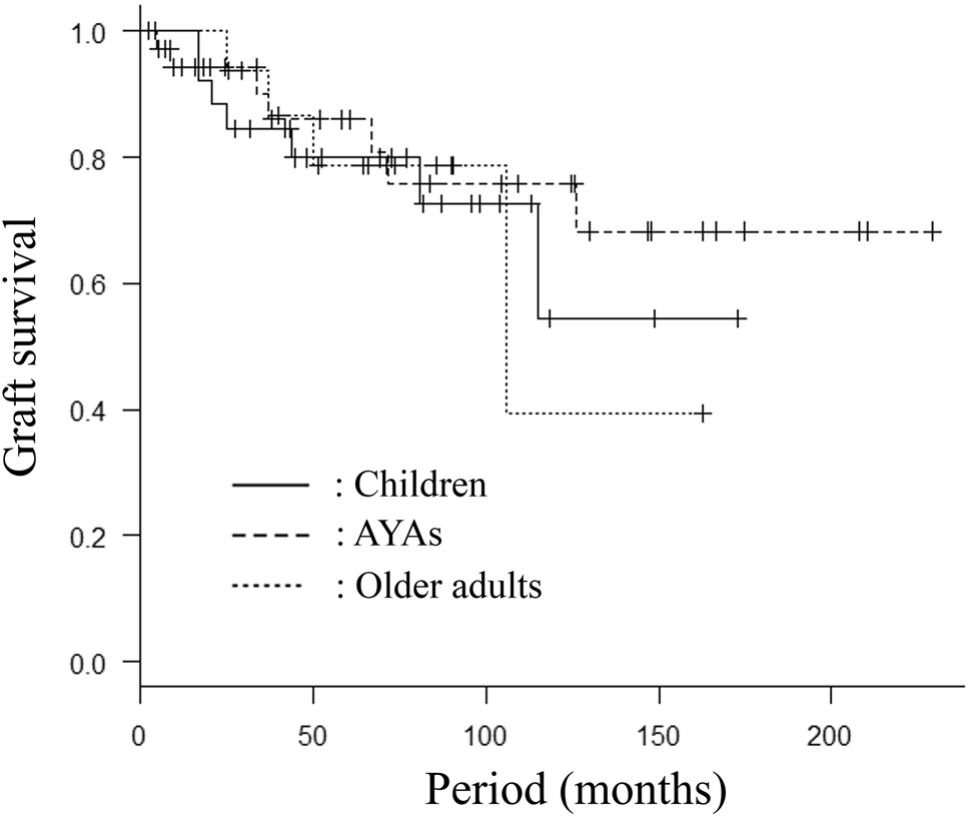
Graft survival (GS) in the three age group patients who underwent frozen autograft reconstruction. The 5- and 10-year GS rates in AYA patients (n = 37) were 86% and 76%, while those in children (n = 27) and older adult patients (n = 16) were 80% and 55%, and 79% and 39%, respectively (*p* = 0.79).

The 5- and 10-year GS rates in AYA patients were 86% and 76%, while those in children and older adult patients were 80% and 55%, and 79% and 39%, respectively (Fig. 1). Total graft removal was performed in 7 AYA cases (19%), while the graft was totally removed in 7 children (26%) and 4 older adult patients (25%), respectively (Table 2). The most common reason for graft removal in AYA patients was infection (n = 5) (14%), and the other reasons for graft removal were recurrence in 2 cases (5%). The reasons for graft removal in the children and older adult patients are shown in Table 2.

**Figure 2:**
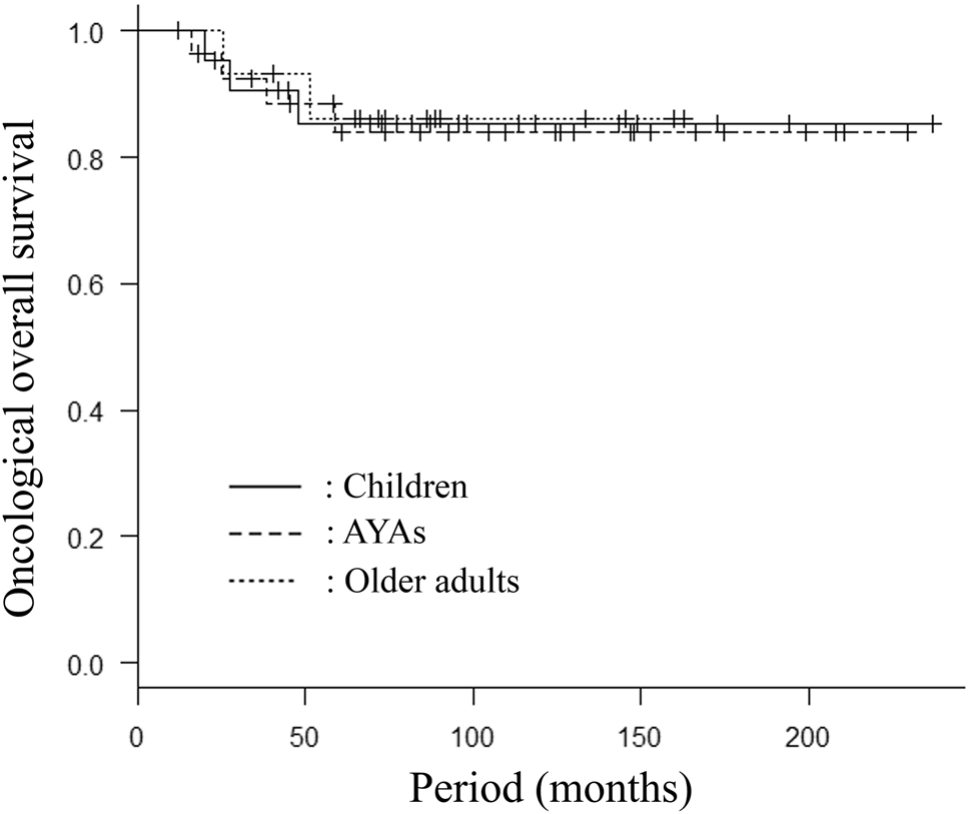
Overall survival (OS) in the three age group patients who underewent frozen autograft reconstruction. Excluding the cases with distant metastasis observed at the first visit, the 5-year and 10-year OS rates in AYA patients (n = 27) were 84% and 84%, while those in children (n = 5) and older adult patients (n = 15) were 85% and 85%, and 86% and 86%, respectively (*p* = 0.98).

**Figure 3:**
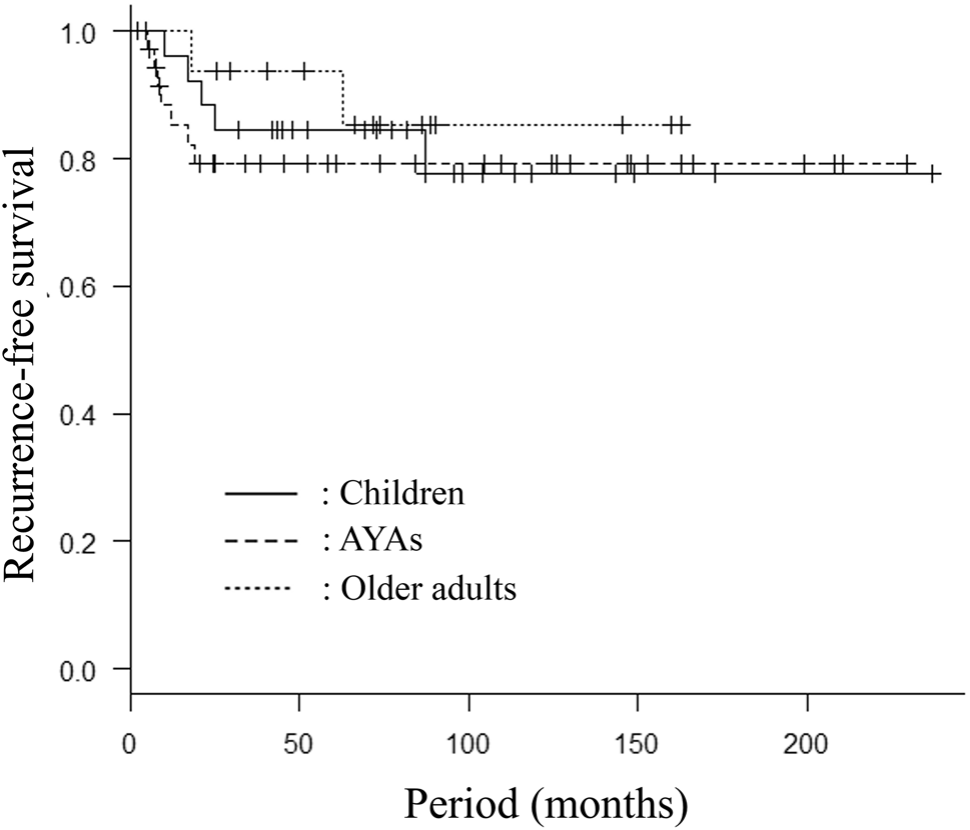
Recurrence-free survival (RFS) in the three age group patients who underwent frozen autograft reconstruction. The 5- and 10-year RFS rates in AYA patients (n = 37) were 79% and 79%, while those in children (n = 27) and older adult patients (n = 16) were 85% and 78%, and 94% and 85%, respectively (*p* = 0.72).

Distant metastasis from sarcoma were observed at the first visit in 9 AYA cases (24%). Excluding these cases, the 5-year and 10-year OS rates in AYA patients were 84% and 84%, while those in children and older adult patients were 85% and 85%, and 86% and 86%, respectively (Fig. 2). Histological examination revealed recurrence in 7 AYA cases (19%), while recurrence was observed in 5 pediatric patients (19%) and 2 older adult patients (13%), respectively. All instances of recurrence arose from the surrounding soft tissue of the frozen autograft, not in the autograft itself. The 5- and 10-year recurrence-free survival (RFS) rates in AYA patients were 79% and 79%, while those in children and older adult patients were 85% and 78%, and 94% and 85%, respectively (Fig. 3).

### Postoperative complications

Infection was most frequently observed postoperative complication in AYA patients, occurring in 7 cases (19%), followed by osteoarthritic change (n = 5; 14%), bone absorption (n = 3; 8%), fracture (n = 2; 5%), nonunion (n = 1; 3%), and lower limb deformity (n = 1; 3%) (Table 2). The sites of infection included the proximal tibia (n = 4), distal tibia (n = 2), and proximal humerus (n = 1), while the site of infection in pediatric patient was distal femur (n = 1) and that in older patients was distal femur (n = 1) and proximal tibia (n = 2). Infection was confirmed at a mean period of 5 months (range, 1–22 months) after the primary surgery in AYA patients. In the 5 cases with infection, the whole frozen autograft was finally removed to suppress the infection completely after conservative treatment. The other two cases recovered from infection following hardware removal and the administration of antibiotics. On the other hand, all of the five AYA patients with osteoarthritic change had undergone osteoarticular reconstruction; among them, three cases required prosthetic joint replacement at a mean period of 18 months after the primary surgery (range, 14–24 months), although the whole frozen autograft was not removed. The other cases were asypmtomatic, and were observed conservatively. Osteoarthritic change was also observed in 4 pediatric patients who underwent osteoarticular reconstruction. Bone absorption was observed at the proximal humerus (n = 2) and calcaneus (n = 1); one case required an additional surgical procedure due to protrusion of the implant, while the others were managed conservatively. One pediatric patient and 2 older adult patients had bone absorption at proximal humerus. The fracture site was major trochanter of the femur and distal tibia in one AYA patient each, and the fracture occurred at 12 and 16 months after surgery, respectively. Both cases underwent osteosynthesis with bone graft and could achieve bone union. One case with nonunion was conservatively observed, and bone union was achieved at 30 months after surgery. Fracture was also observed in 8 pediatric patients and 1 older adult patient. One AYA patient with lower limb deformity underwent correction of the deformity using external fixation at 7 years after the primary surgery, while 5 pediatric patients had lower limb deformity including limb length discrepancy. Amputation for salvage of refractory infection and soft tissue recurrence involved with neurovascular bundles was performed in three and two AYA patients, respectively. Salvage amputation was performed in a total of 3 pediatric patients and 1 older adult patient. The postoperative complications with each freezing procedure in AYA patients and the other two age groups are shown in Tables 3 and 4.

**Table 3:** The clinical outcomes and complications in the patients who underwent free freezing procedure on the appendicular skeleton in the three age groups.

Generation	Children (≤ 14 years old)	AYAs (15 to 39 years old)	Older adults (≥ 40 years old)	*P*-value
Case (n)	11	21	5	–
Chemotherapy (n) (%)	11	19	4	0.14
Oncological outcome (n) (%)	CDF 6 (54%)	CDF 8 (38%)	CDF 2 (40%)	0.81
	NED 3 (27%)	NED 6 (29%)	NED 1 (20%)	
	AWD 1 (9%)	AWD 1 (5%)	AWD 1 (20%)	
	DOD 1 (9%)	DOD 6 (29%)	DOD 1 (20%)	
Median period of bone union (mos.) (range)	8 (4–15)	8 (3–21)	11 (7–15)	0.47
Bone union rate within 1 year	89%	93%	50%	0.12
Functional score (MSTS score) (n) (%)	Excellent 8 (89%) Good 1 (11%) Fair 0 (0%) Poor 0 (0%)	Excellent 14 (78%) Good 3 (17%) Fair 1 (6%) Poor 0 (0%)	Excellent 5 (100%)	1
5-year graft survival (%)	64%	83%	75%	0.64
**Reasons for graft removal**				
Infection (n) (%)	1 (9%)	4 (19%)	1 (20%)	0.84
Fracture (n) (%)	1 (9%)	0 (0%)	0 (0%)	0.43
Non-union (n) (%)	0 (0%)	0 (0%)	0 (0%)	1
Osteoarthritic change (n) (%)	1 (9%)	0 (0%)	0 (0%)	0.43
Recurrence (n) (%)	2 (18%)	2 (10%)	1 (20%)	0.51
**Complications**				
Infection (n) (%)	1 (9%)	5 (24%)	1 (20%)	0.84
Fracture (n) (%)	2 (18%)	0 (0%)	1 (20%)	0.1
Non-union (n) (%)	1 (9%)	1 (5%)	0 (0%)	1
Limb deformity (n) (%)	4 (36%)	1 (5%)	0 (0%)	0.04
Osteoarthritic change (n) (%)	2 (18%)	2 (10%)	0 (0%)	078
Bone absorption (n) (%)	0 (0%)	2 (10%)	1 (20%)	0.23
**Salvage amputation**				
Recurrence (n) (%) Infection (n) (%)	1 (9%) 1 (9%)	2 (10%) 3 (14%)	1 (6%) 0 (0%)	0.78 1.00

*CDF* clinically disease-free, *NED* no evidence of disease, *AWD* alive with disease, *DOD* dead of disease, *DOOD* dead of other diseases, *MSTS* Musculoskeletal Tumor Society.

**Table 4:** The clinical outcomes and complications in the patients who underwent pedicle freezing procedure on the appendicular skeleton in the three age groups.

Generation	Children (≤ 14 years old)	AYAs (15 to 39 years old)	Older adults (≥ 40 years old)	*P*-value
Case (n)	16	16	11	–
Chemotherapy (n)	16	14	4	< 0.01
Oncological outcome (n) (%)	CDF 10 (63%)	CDF 8 (50%)	CDF 8 (72%)	0.46
	NED 2 (13%)	NED 0 (0%)	NED 0 (0%)	
	AWD 1 (6%)	AWD 4 (25%)	AWD 2 (18%)	
	DOD 3 (19%)	DOD 4 (25%)	DOOD 1 (9%)	
Median period of bone union (mos.) (range)	5 (3–7)	7 (4–9)	10 (4–24)	0.32
Bone union rate within 1 year	100%	100%	75%	0.46
Functional score (MSTS score) (n) (%)	Excellent 10 (83%) Good 1 (8%) Fair 1 (8%) Poor 0 (0%)	Excellent 9 (82%) Good 1 (9%) Fair 1 (9%) Poor 0 (0%)	Excellent 8 (80%) Good 1 (10%) Fair 1 (10%) Poor 0 (0%)	1
5-year graft survival (%)	92%	89%	80%	0.65
**Reasons for graft removal**				
Infection (n) (%)	0 (0%)	1 (6%)	2 (18%)	0.25
Fracture (n) (%)	0 (0%)	0 (0%)	0 (0%)	1
Non-union (n) (%)	0 (0%)	0 (0%)	0 (0%)	1
Osteoarthritic change (n) (%)	1 (6%)	0 (0%)	0 (0%)	1
Recurrence (n) (%)	1 (6%)	0 (0%)	0 (0%)	1
**Complications**				
Infection (n) (%)	0 (0%)	2 (13%)	2 (18%)	0.28
Fracture (n) (%)	6 (38%)	2 (13%)	0 (0%)	0.06
Non-union (n) (%)	2 (13%)	0 (0%)	1 (9%)	0.46
Limb deformity (n) (%)	1 (6%)	0 (0%)	0 (0%)	1
Osteoarthritic change (n) (%)	2 (13%)	3 (19%)	0 (0%)	0.41
Bone absorption (n) (%)	1 (6%)	1 (6%)	1 (9%)	1
**Salvage amputation**				
Recurrence (n) (%) Infection (n) (%)	1 (6%) 0 (0%)	0 (0%) 0 (0%)	0 (0%) 0 (0%)	1 1

*CDF* clinically disease-free, *NED* no evidence of disease, *AWD* alive with disease, *DOD* dead of disease, *DOOD* dead of other diseases, *MSTS* Musculoskeletal Tumor Society.

### The postoperative function

In the 17 AYA cases in which the data were available, bone union was achieved after a mean period of 8 months (range, 3–21 months), while that was a mean period of 6 months (range, 3–15 months) in pediatric patients and a mean period of 10 months (range, 4–24 months) in older adult patients, respectively (Table 2). Among the 17 AYA cases, 16 (94%) achieved bone union within one year after primary surgery, while 94% (16/17) in pediatric patients and 63% (5/8) in older adult patients achieved bone union within one year after primary surgery, respectively (Table 2). The MSTS score at the latest follow-up was excellent in 23 (79%) of the 29 AYA patients for whom the data were available, while 86% (18/21) in pediatric patients and 87% (13/15) in older adult patients, respectively, were excellent according to MSTS score (Table 2). The postoperative function with each freezing procedure in the three age groups are shown in Tables 3 and 4.

## Discussion

We investigated the clinical outcomes and complications of frozen autograft reconstruction for the treatment of primary bone sarcoma on the appendicular sites in AYA patients, and compared the findings with those for children and older adult patitents. In addition to good clinical outcomes, a high rate of early bone union and graft survival were observed in AYA patients in this study. Nonunion, fracture, and limb deformity were infrequently observed, however, there were some complications, including infection and osteoarthritic change.

Reconstruction using devitalized tumor-bearing autografts after bone tumor excision is well-known approach in orthopedic oncology^17–32^. Various devitalizing methods are available today, including irradiation^17–20^, pasteurization^21–23^, and freezing^24–32^. Reconstruction using any devitalized autograft received coverage from the social insurance system in Japan in April 2020; the medical expenses borne by patients are now lower, and the surgical procedures can be performed at any hospital in Japan. Numerous investigations of each method of autograft reconstruction, irrespective of age, have been reported in previous studies^17–32^. The autograft can fit perfectly with the original site, and the retracted tendon or muscles can be reattached^29,30^. However, no studies have described the clinical outcomes of autograft reconstruction focusing on skeletally mature AYAs.

In our study, bone union at the osteotomy site between the frozen autograft and the host bone was achieved with a rate of 94% within 1 year after surgery in AYA patients, which was higher and earlier than those values in older adult patients (63%) (Table 2). The fracture and nonunion rates (5%; 2/37, and 3%; 1/37) in AYA patients were lower than those in pediatric patients (30%; 8/27, and 11%; 3/27) (Table 2). The bone quality and mechanical loading such as weight-bearing greatly influences bone healing^34–37^. AYA patients have fewer comorbidities including osteoporosis, diabetes mellitus, or a smoking habit, than older adult patients^34,35^, and AYA patients can adhere better to the limited weight-bearing after surgery than pediatric patients^36,37^. These characteristics in AYA patients might contribute to the good bone healing and the occurrence of fewer complications than in younger patients. Furthermore, bone morphogenetic proteins (BMPs), such as BMP-2 or BMP-7, was retained in the frozen bone, and has osteoconductive or osteoinductive properties, even after freezing, according to the findings of previous studies^38–40^. Our data also suggested that these proteins were well preserved and structural strength was maintained in the frozen bone in skeletally mature AYA patients, so bone union could be achieved at an early point and bone fracture might occur infrequently. However, further investigation is needed to confirm the early restoration of these properties in the frozen bone of AYA patients.

The GS in AYA patients was higher than that in the other two age groups, although no significant differences were observed (Fig. 1). Longevity could be achieved, which was the primary aim of surgery for AYA patient with bone tumors. The most common reasons for graft removal in the present study were infection and recurrence, as was described in previous studies^17–32^. Whole graft removal due to infection was performed in 5 AYA patients (14%); the sites of infection were the tibia in all 5 patients. The site of tibia was reported to be susceptible to occur infection in surgery for bone tumors^10,16,41^. In the present study, a total of 8 patients (30%, 8/27) had had infection on the site of tibia (pediatric; 0/10 [0%], AYA; 6/11 [54%], older adult; 2/6 [33%]). Free freezing procedure and pedicle freezing procedure were perfomed in five and three patients. Five patients had undergone the osteoarticular reconstruction, two had undergone the prosthesis-composite reconstruction, and the remaining one had undergone intercalary recostruciton. Intrameullary nail and plate for fixation, and prosthesis were used in 4, 2, and 2 patients, respectively. While the reasons for such a high rate of infection on the site of tibia were not clear, potential reasons include a small amount of soft tissue coverage on the tibia, the large bone size for osteoarticular reconstruction in five cases, and a number of different operation devices being used for intramedullary nail fixation in four cases. Our data suggested that sufficient amounts of antibiotics for a longer period of time after surgery—especially in cases involving tumors of the tibia—should be administered in order to prevent infection in adult patients including AYA and older adult patients. Little is known about the difference in the restoration of the immune system in devitalized bone according to age, so further investigations will be necessary to confirm the restoration of the frozen bone marrow, which have an ability to generate immune cells for bacteria^42–44^.

The recurrence rate was reported to be 6–40% after completing initial treatment, depending on the surgical margin, tumor sites, and efficacy of chemotherapy^10,45,46^. In our study, all the recurrence in 7 AYA cases (19%; 7/37) arose from the soft tissue surrounding the frozen autograft, and not from the autograft itself, as was described in previous studies^24–29^. The rates were not significantly higher than those in children and older adult patitens (19%; 5/27, and 13%; 2/16). Graft removal due to soft tissue recurrence could not help being performed as a radical treatment, despite the fact that neither any tumors nor complications were involved with the graft. The surgical procedure included amputation in 2 cases. All the soft tissue recurrence in the other 5 cases was excised with a wide margin after adjuvant chemotherapy, preserving the intact frozen bone.

The rate of postoperative osteoarthritic change after reconstruction of osteoarticular allograft, or osteoarticular frozen autograft including total epiphysis, were reported to be 39–100% in the previous studies^15,31,47^. In our study, osteoarthritic change was observed in 5 of the 10 (50%) AYA patients who had underwent osteoarticular frozen autograft reconstruction, while 4 of the 5 pediatric patients (80%) and 0 of the 2 older adult patients (0%) developed osteoarthritic changes after osteoarticular frozen autograft reconstruction (Table 2). The advantage of osteoarticular reconstruction is that it has the potential to preserve the joint, especially in young patients; however, high rates of joint degeneration were reported, and these patients eventually require prosthetic joint replacement at skeletal maturity^15,31,47^. The cartilage is usually thick in children and no degenerative change was observed in skeletally mature AYAs unless the cartilage is affected by a bone tumor; however, the cartilage was reported to be degenerated by freezing methods in previous studies^48^. Our data suggested that the frozen osteoarticular bone in pediatric and AYA patients, which is composed of large amount of chondrocytes, might be fragile especially with regard to articular cartilage of the distal femur or proximal tibia. Three patients underwent prosthetic joint replacement because of the progression of degenerative change. Close examinations during regular follow-up are necessary to detect the progression of osteoarthritic change and the timing of reoperation, such as total knee joint surface replacement, especially in young patients with osteoarticular reconstruction.

No cases of growth disorder of limb length discrepancy was observed among the AYA patients in this study, although involvement of physis was observed in 8 cases. This was due to the closure of the physis after the growth of bone had been almost completed in the AYA patients. In the present study, limb length discrepancy ≥ 3 cm was observed only in 5 pediatric cases (19%, 5/27), and all the cases underwent limb lengthening using external fixation.

Bone absorption was observed in 3 AYA patients, 1 pediatric patient and 2 older adult patients (Table 2). The incidence rate was similarly low in all three age groups. 5 of six such patients in this study showed bone absorption in proximal humerus, a site of bone absorption that tended to occur in previous studies^27^, and the cause might be due to non-weight bearing location.

The functional outcome at the latest follow-up examination was excellent in most AYA cases with a rate of 79% (23/29), which was higher than that with endoprosthesis reconstruction in previous studies^9–12^. The MSTS scores were excellent in 11 of the 12 available cases, even with prosthesis-composite reconstruction in AYA patients. This was because biologicacl reconstruction was performed rather than endoprosthesis replacement in order to retain as normal a joint function as possible (including reattachment of surrounding soft tissues of capsules, tendon, and muscles).

The present study was associated with some limitations. First, this was a retrospective study of a relatively small study population in a single institution, although our hospital is a high-volume sarcoma center. Second, each age group included heterogeneous characteristics due to the rarity of this diseases, although there were no statistically significant differences in the variables observed in the three age groups. Third, a few cases were only followed for a short time, because StageIV patients who underwent surgical procedures were also included in the present study. Fourth, the timing of bone union might not have been accurately determined because of the different follow-up intervals. Fifth, the frozen bone size and amount of soft tissue coverage on the frozen bone differed among cases.

In conclusion, frozen autograft reconstruction in skeletally mature AYA patients was associated with good clinical outcomes and excellent function. Long-term graft survival, and early bone union was achieved, compared with those of older adult patients, while only a few cases had had fracture, nonunion, or limb deformity, in comparison to those of pediatric patients. In addition to monitoring the oncological course in all generation, care is required during follow-up to prevent infection at the site of the tibia in AYA and older adult patients, and to detect osteoarthritis after osteoarticular reconstruction in pediatric and AYA patients.

## Methods

### Patient enrollment

A total of 146 patients underwent reconstruction using a frozen autograft treated with liquid nitrogen after excision for soft tissue and bone tumors at our hospital from 1999 to 2016. Among them, 80 patients with primary bone sarcoma on the appendicular skeleton were enrolled in the present study, and the study population included 27 children ≤ 14 years old, 37 AYAs 15 to 39 years old^2^, and 16 older adults ≥ 40 years old. This study was conducted in accordance with the 1975 Declaration of Helsinki. The study was approved by the Ethical Institutional Review Board of Kanazawa University Hospital (2019-061 [3094]), and written informed consent was obtained from all of the study participants.

### Patient characteristics’ data collection

The age, sex, tumor location (upper extremities or lower extremities), histological types (2013 WHO classification^1^), staging (AJCC 8th edition^33^), surgical techniques for freezing (pedicle freezing or free freezing), reconstruction methods (intercalary reconstruction, osteoarticular reconstruction and prosthesis-composite reconstruction), and postoperative follow-up periods, were retrospectively investigated in the three different age groups, using medical records. Surgical procedures were determined, basically depending on tumor location. Firstly, in the case of a tumor located on the center of the bone of the proximal humerus, proximal femur, proximal tibia, or distal tibia, the pedicle freezing procedure was usually applied. Then, in the case of a tumor located on the center of the bone of the distal femur, or other sites, the free freezing procedure was selected. In the case of a tumor located on either side of the bone, hemocortical bone excision and free freezing procedure was often performed, however, in the present study, the patients who underwent this procedure were excluded because it was considered to be a confounding factor due to the advantage of early bone union. The reconstruction methods were decided based on tumor infiltration to the epiphysis of the long bone. When the tumor did not infiltrate the epiphysis of the bone, the intercalary reconstruction method was usually applied. When the tumor infiltrated the epiphysis, the osteoarticular reconstruction method or autograft-prosthesis composite reconstruction method was selected.

### Patient follow-up and assessment

The postoperative images were regularly examined in the hospital setting until patients had completed adjuvant chemotherapy and been discharged at about six months after surgery. The patients were observed at follow-up intervals of one to two months at the outpatient clinic. One year after surgery, provided there had been no adverse events or complications, the patients were followed up every 4 months during the first 5 years after treatment completion and every 6–12 months thereafter. The routine assessment includes magnetic resonance imaging (MRI) for detection of local recurrence, and systemic computed tomography (CT) and chest X-ray for detecting distant metastasis for the first five years. Thallium scintigraphy, and positron emission tomography is performed annually or when necessary. The graft survival (GS), overall survival (OS), recurrence-free survival (RFS), the period until bone union, and postoperative complications in AYA patients were evaluated and compared with those in children and older adults patients according to the freezing procedure (free freezing or pedicle freezing). With the exception of pedicle frozen autograft-prosthesis composite reconstruction cases and cases that were to evaluate radiographically due to the hardware, bone union was defined by the observation of a bridging callus on two different X-rays or on CT within two years after primary surgery from the available data. Postoperative complications included infection, fracture, non-union, osteoarthritic change, limb deformity (including limb length discrepancy), bone absorption. Non-union was defined as no achievement of bone union after two years had passed postoperatively. Infection was defined based on a positive culture and clinical symptoms after primary surgery. Osteoarthritic change was defined as a progression of the grade of osteoarthritis as evaluated by the Kellgren-Lawrence classification. Limb deformity was defined as a severe varus/valgus deformity, or limb length discrepancy ≥ 3cm that disturbed daily life. GS was defined as the period until the removal of the whole frozen autograft, excluding cases in which the frozen autograft was partially removed or removed due to soft tissue recurrence around the intact frozen bone. The reasons for graft removal were also investigated. OS was defined as the postoperative period until the death of the patient with primary bone sarcoma. RFS was defined as the period until histological examination of local recurrence. The functional outcomes at the latest follow-up examination were assessed based on the available data using the Musculoskeletal Tumor Society (MSTS) score after the exclusion of deceased cases.

### Surgical procedures

**Figure 4:**
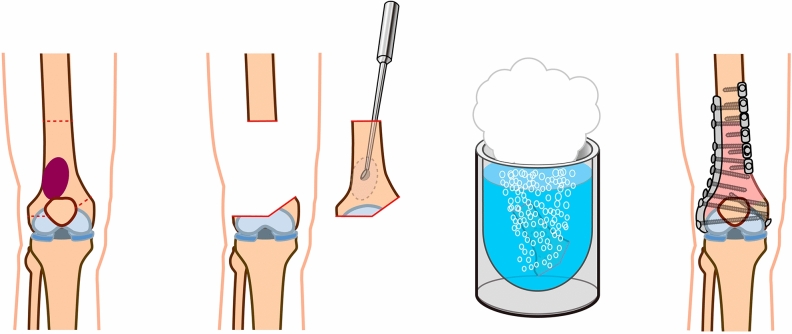
Free freezing procedure. The bone tumor is excised *en bloc* with an adequate margin. After curettage inside the bone specimen and removal of the surrounding soft tissue, the bone specimen was immersed and frozen for 20 min in liquid nitrogen. After thawing, the devitalized bone was fixed to the residual healthy bone on the original site with double locking plates.

**Figure 5:**
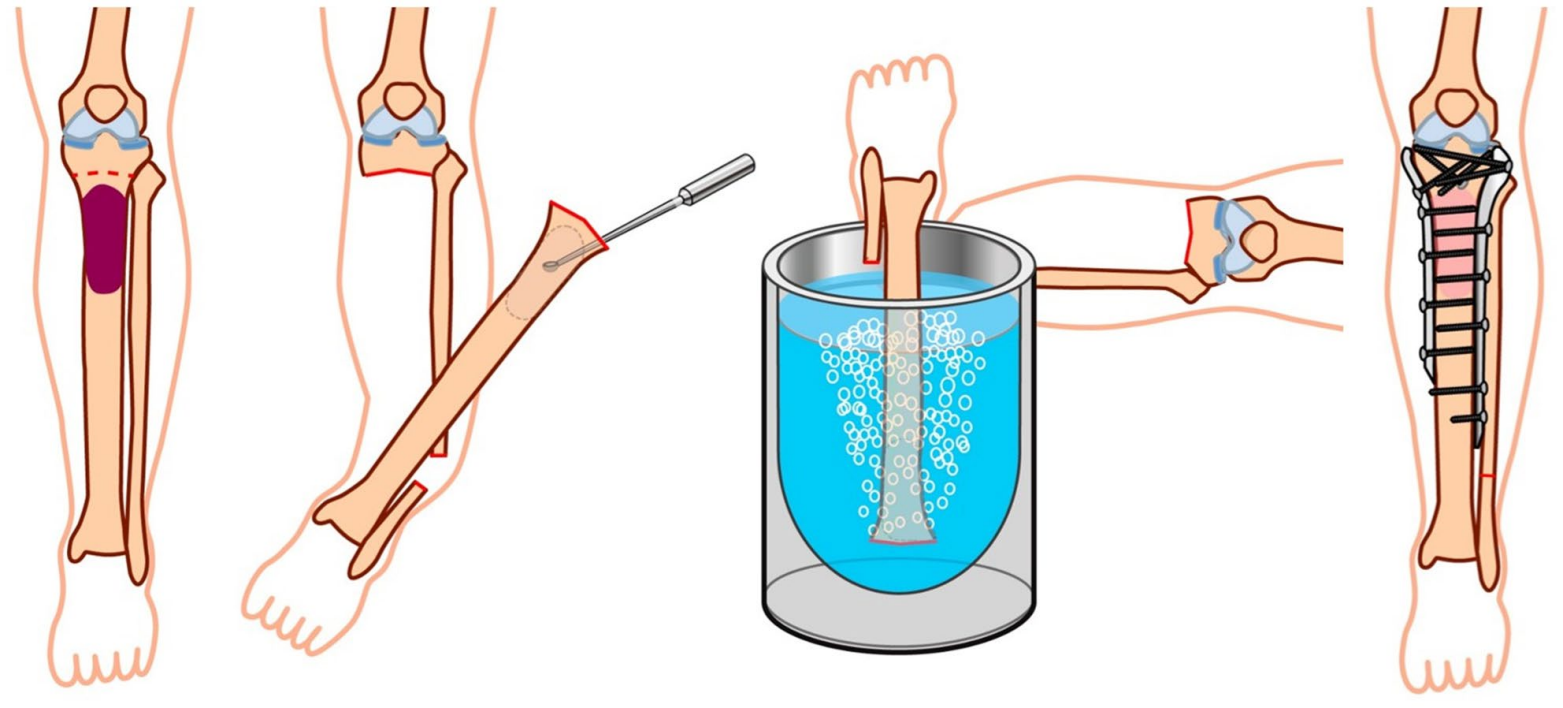
Pedicle freezing procedure. The bone tumor is excised with a pedicle to the healthy bone with an adequate margin. After curettage inside the tumor-bearing bone and removal of the surrounding soft tissue, the remaining bone was turned upside down, and then immersed and frozen for 20 min in liquid nitrogen. After thawing, the devitalized bone was fixed to the residual healthy bone on the original site with double locking plates.

The bone tumor was excised *en bloc* (Free freezing procdure, Fig. 4) or with a pedicle to the healthy bone (Pedicle freezing procedure, Fig. 5) approximately 3 cm from the border of the tumor using a microsurgical saw or T-saw. The excised specimen’s soft tissues were removed on another operation table or with the healthy tissues being carefully protected using surgical sheets, and the tumor inside the bone was curetted before freezing. The curetted bone was frozen for 20 min in liquid nitrogen that was stored in sterilized flask, and then thawed at room temperature for 15 min, and in a solution of 0.3% iodine and distilled water for another 15 min. The frozen autograft was fixed to the residual healthy bone on the original site with double or triple locking plates, or intramedullary nail, or with a composite prosthesis using polymethyl methacrylate. The surgical procedures were not changed according to the histological subtype.

### Statistical analyses

Between the three groups, one-way ANOVA test for follow-up periods and bone union periods, and a chi-squired test for sex, tumor location, stage, histological subtypes, freezing procedures, reconstruction methods, chemotherapy, oncological outcomes, bone union rates, functional outcomes, each reason for graft removal, each complication, and amputation were compared. In both tests, *P*-values of < 0.05 was considered to indicate statistical significance. The GS, OS, and RFS, were plotted using Kaplan–Meier curves, and Bonferonni’s test was used to compare these survival rates among the three age groups. *P* values of < 0.05 were considered to indicate statistical significance. All statistical analyses were performed using EZR (Saitama Medical Center, Jichi Medical University, Saitama, Japan), which is a graphical user interface for the R software program (The R Foundation for Statistical Computing, Vienna, Austria)^49^.

### Ethical approval

This study was conducted in accordance with the 1975 Declaration of Helsinki.

### Approval for human experiments

The study was approved by the Ethical Institutional Review Board of the Kanazawa University Hospital (2019-061 (3094)), and written informed consent was obtained from all study participants and/or their parents (in case of patients aged < 18 years old).

### Consent to participate/consent to publish

The consent for partcicipation and publication of the manuscript and the related images from the patients and/or their parents (in case of patients aged < 18 years old) was obtained by the Kanazawa University Hospital.

## Data Availability

All data generated or analyzed during the present study are included in this published article.

## Abbreviations

AYA: Adolescents and young adults
AJCC: American Joint Committee on Cancer
OS: Overall survival
RFS: Recurrence-free survival
GS: Graft survival
MSTS: Musculoskeletal Tumor Society
MRI: Magnetic reasonance imaging
CT: Computed tomography
